# Supplementary material for: Pharmacological activation of the circadian component REV-ERB inhibits HIV-1 replication
Source: Sci Rep. 2020 Aug 6;10:13271. doi: 10.1038/s41598-020-70170-3 (PMC7413328; doi:10.1038/s41598-020-70170-3)
Supplement: Supplementary file 1 — Supplementary Figures. [file 41598_2020_70170_MOESM1_ESM.pdf]

SUPPLEMENTARY MATERIALS for MS:

Pharmacological activation of the circadian component REV-ERB inhibits HIV-1 replication

Helene Borrmann[1*], Rhianna Davies[2*], Matthew Dickinson[1], Isabela Pedroza-Pacheco[1], Mirjam Schilling[1], Alun Vaughan-Jackson[3], Andrea Magri[1], William James[3], Peter Balfe[1] Persephone Borrow[1], Jane A McKeating[1] and Xiaodong Zhuang[1**]

1 – Nuffield Department of Clinical Medicine, University of Oxford, OX3 7FZ, UK.

2 – Institute of Immunity and Immunotherapy, University of Birmingham, B15 2TT. UK.

3 – Sir William Dunn School of Pathology, University of Oxford, OX1 3RE

*Shared first authorship

** Corresponding author: *xiaodong.zhuang@ndm.ox.ac.uk*

Supplementary figure 1

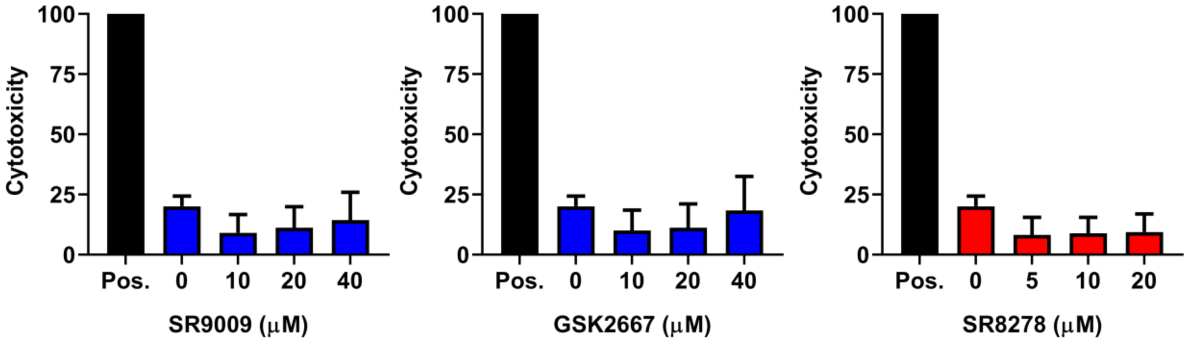

SFig.1 Lack of cytotoxicity of REV-ERB ligands for TZM-bl cells. TZM-bl cells were treated with REV-ERB agonists SR9009 or GSK2667 or the antagonist SR8278 at a range of doses for 24h and cytotoxicity determined using an LDH assay (mean ± S.E.M., n = 2). Data are expressed relative to the positive control representing total cell lysate (100% cytotoxicity).

Supplementary figure 2

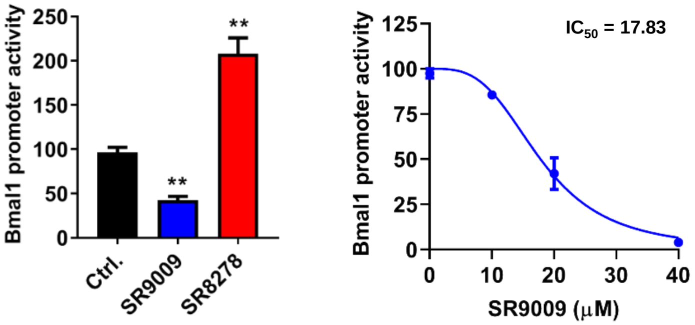

SFig.2 Effect of REV-ERB ligands on Bmal1 promoter activity in Jurkat cells. Jurkat cells stably expressing a Bmal1 promoter-luciferase construct were treated with the REV-ERB agonist SR9009 (20 μM) or antagonist SR8278 (20 μM) and luciferase activity measured after 24h (mean ± S.E.M., n = 3, One-way ANOVA). The IC_{50} of SR9009 was determined at 24h post treatment by quantifying luciferase activity.

Supplementary figure 3

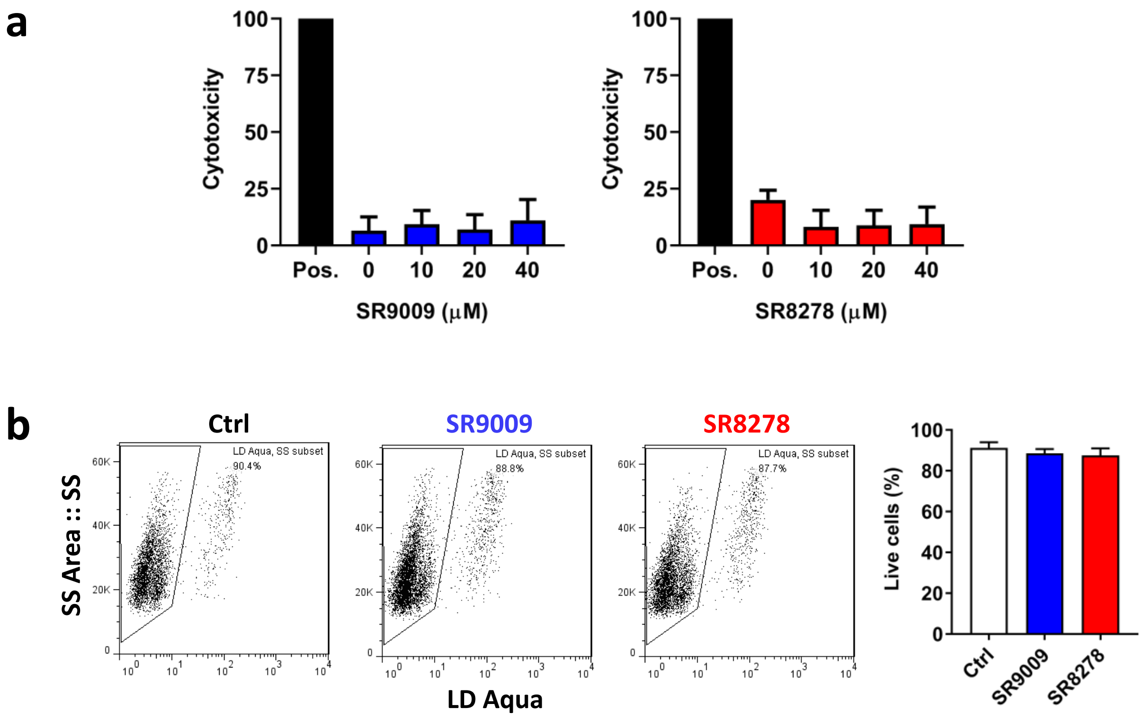

SFig.3 Lack of cytotoxicity of REV-ERB ligands for Jurkat cells. (a) Jurkat cells were treated with the REV-ERB agonist SR9009 or antagonist SR8278 at a range of doses for 24h and cytotoxicity determined using an LDH assay (mean ± S.E.M., n = 2). Data are expressed relative to a positive control representing total cell lysate (100% cytotoxicity). **(b)** Jurkat cells were treated with the REV-ERB agonist SR9009 or antagonist SR8278 for 24h or medium only as a negative control, and viability assessed by flow cytometry using a live-dead stain. Dot plots illustrate staining of one representative biological sample and summary plots of the percentage of live cells are shown on the right (n=7, mean + S.E.M., One-way ANOVA).

Supplementary figure 4

a

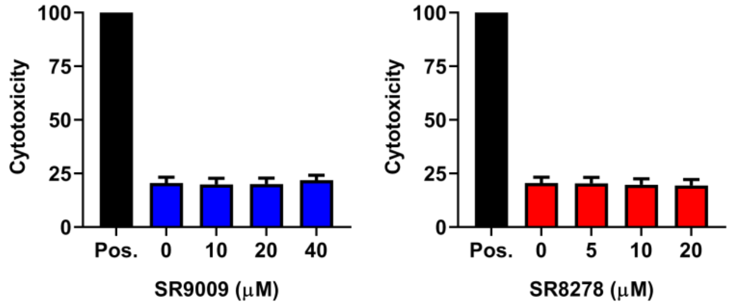

b

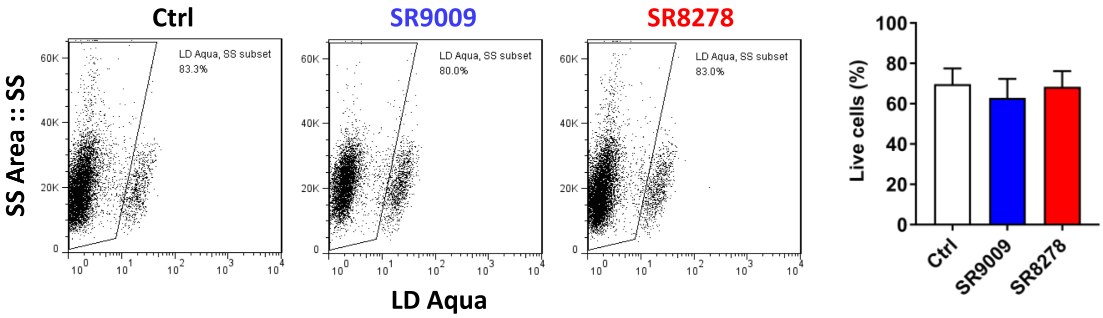

SFig.4 Lack of cytotoxicity of REV-ERB ligands for human primary CD4 T cells. Primary human CD4 T cells were activated with anti-CD3/CD28 for 3 days and treated with the REV-ERB agonist SR9009 or antagonist SR8278 at a range of doses for 24h and cytotoxicity determined using an LDH assay (mean ± S.E.M., n = 3). Data are expressed relative to the positive control representing total cell lysate (100% cytotoxicity). **(b)** Activated CD4 T cells were treated with the REV-ERB agonist SR9009 or antagonist SR8278 for 24h or medium only as a negative control, and cell viability assessed by flow cytometry using a live-dead stain. Dot plots illustrates staining of one representative biological sample and summary plots of the percentage of live cells are shown on the right (n=7, mean + S.E.M., One-way ANOVA).

Supplementary figure 5

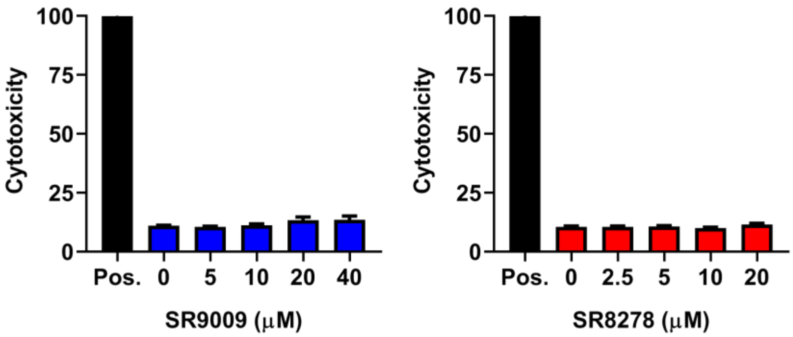

SFig.5 Lack of cytotoxicity of REV-ERB ligands for human induced pluripotent stem cells (iPSCs) derived macrophages. Human iPSCs-derived macrophages were treated with the REV-ERB agonist SR9009 or antagonist SR8278 at a range of doses for 24h and cytotoxicity determined using a LDH assay (mean ± S.E.M., n = 3). Data are expressed relative to the positive control representing total cell lysate (100% cytotoxicity).

Supplementary figure 6

ROR response element (Start 412 bp)

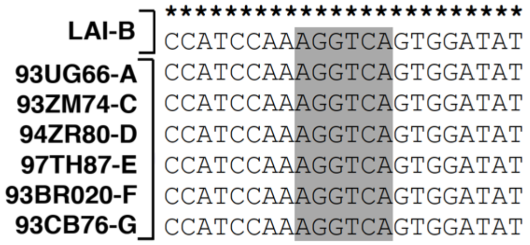

Glucocorticoid receptor elements (start: 343 bp)

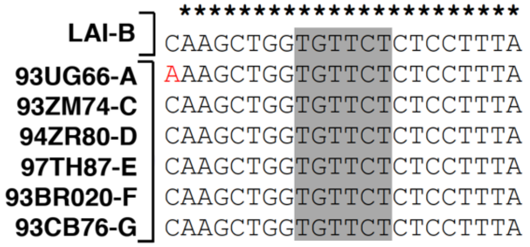

SFig.6 Conserved ROR response element and glucocorticoid receptor element in the HIV-1 LTR.

Supplementary figure 7

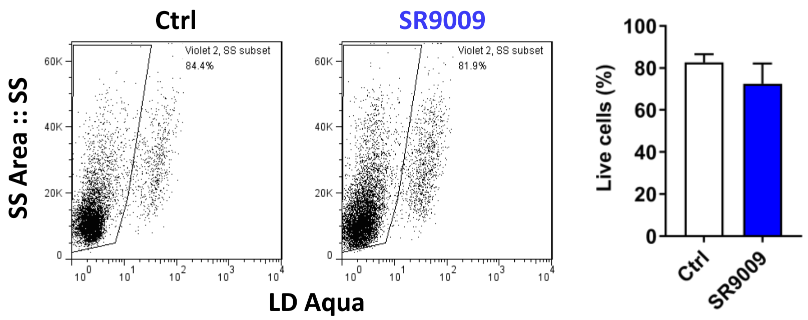

SFig.7 Viability of peripheral blood mononuclear cells (PBMCs) following extended treatment with SR9009. Human PBMCs were depleted of CD8 T cells and activated by culture with antibodies to CD3 and CD28 for 3 days, then infected with HIV-1 and cultured in medium with or without SR9009 (20 μM) for a further 7 days. The first biological repeat employed cells from a single HIV-seronegative donor, whilst repeats two and three employed cells pooled from three healthy donors. Cell viability was assessed by flow cytometry using a live-dead stain on day 7 post SR9009 treatment. Dot plots illustrate staining of one representative biological sample and summary plots of the percentage of live cells are shown on the right (n=3, mean + S.E.M., paired t test).
